# Aurora Kinase A inhibition enhances DNA damage and tumor cell death with ^131^I-MIBG therapy in high-risk neuroblastoma

**DOI:** 10.21203/rs.3.rs-3845114/v1

**Published:** 2024-01-18

**Authors:** Prerna Kumar, Jessica Koach, Erin Nekritz, Sucheta Mukherjee, Benjamin S. Braun, Steven G. DuBois, Nicole Nasholm, Daphne Haas-Kogan, Katherine K. Matthay, William A. Weiss, Clay Gustafson, Youngho Seo

**Affiliations:** 1. University of Illinois College of Medicine at Peoria, Department of Pediatrics, Peoria, IL, United States; 2. University of California San Francisco, San Francisco, CA, United States; 3. Dana-Farber/Boston Children’s Cancer and Blood Disorders Center, Harvard Medical School, Boston, MA, United States; 4. Department of Radiation Oncology, Dana-Farber Cancer Institute, Brigham and Women’s Hospital, Harvard Medical School, Boston, MA, United States; 5. University of California San Francisco, Departments of Neurology, Neurosurgery, and Brain Tumor Research Center, San Francisco, CA, United States; 6. Department of Radiology and Biomedical Imaging, University of California, San Francisco, CA, United States; 7. UCSF Helen Diller Family Comprehensive Cancer Center, San Francisco, CA, United States

**Keywords:** neuroblastoma, Aurora Kinase A inhibitors, ^131^I-MIBG, metaiodobenzylguanidine, radiopharmaceutical

## Abstract

**Background:**

Neuroblastoma is the most common extra-cranial pediatric solid tumor. ^131^I-metaiodobenzylguanidine (MIBG) is a targeted radiopharmaceutical highly specific for neuroblastoma tumors, providing potent radiotherapy to widely metastatic disease. Aurora kinase A (AURKA) plays a role in mitosis and stabilization of the MYCN protein in neuroblastoma. Here we explore whether AURKA inhibition potentiates a response to MIBG therapy.

**Results:**

Using an *in vivo* model of high-risk neuroblastoma, we demonstrated a marked combinatorial effect of ^131^I-MIBG and alisertib on tumor growth. In *MYCN* amplified cell lines, the combination of radiation and an AURKA A inhibitor increased DNA damage and apoptosis and decreased MYCN protein levels.

**Conclusion:**

The combination of AURKA inhibition with ^131^I-MIBG treatment is active in resistant neuroblastoma models and is a promising clinical approach in high-risk neuroblastoma.

## Introduction and Background

Neuroblastoma, a tumor of the sympathetic nervous system, is the most common extra-cranial pediatric solid tumor. High-risk disease accounts for approximately half of all initial presentations and 15% of pediatric cancer related mortality [1]. Neuroblastoma has two unique vulnerabilities - selective uptake of metaiodobenzylguanidine (MIBG) and frequent amplification of the *MYCN* oncogene.

Neuroblastoma is a radio-sensitive tumor thus external beam radiation is a critical part of the current standard of care [2]. MIBG is a norepinephrine analog that, when radiolabeled with I-131, provides selective radiation therapy via uptake by the human norepinephrine transporter (hNET), which is widely expressed on the neuroblastoma cell surface [3]. ^131^I-MIBG is an active agent used to treat relapsed and refractory neuroblastoma and is currently being studied in newly diagnosed patients with high-risk disease in a randomized Phase 3 trial (NCT03126916) and as Phase 2 induction therapy [4]. MIBG has a reported response rate of 25–40% in relapsed and refractory disease [5,6]. We hypothesized that, if MIBG were to be given in combination with targeted radio-sensitizing therapies, this response rate could be improved.

We have previously investigated the radio-sensitizing effect of vorinostat, a histone deacetylase (HDAC) inhibitor, in neuroblastoma. When treated with external beam radiation and drug, tumor cells showed increased cell death *in vitro* and decreased tumor growth *in-vivo* [7]. Vorinostat treated tumors showed reduced levels of Ku-86, a DNA repair enzyme, that potentiates the effect of radiation in cultured neuroblastoma cells [7]. Vorinostat treatment also increases the expression of hNET, the main transporter of MIBG, in neuroblastoma cells [8]. Subsequent clinical trials combining vorinostat and ^131^I-MIBG, showed combinatorial efficacy with improved responses compared to MIBG alone [9]. This successful translation of a targeted therapy in combination with ^131^I-MIBG from pre-clinical models to clinical trials is encouraging for the development of additional synergistic agents to further improve response rates.

*MYCN* is a transcription factor oncogene and known driver of neuroblastoma associated with high-risk disease and poor overall survival. As such, *MYCN* is a tempting therapeutic target; however, directly inhibiting *MYCN* is challenging since it is not an easily druggable enzyme. In addition, as a transcription factor important in cell division, *MYCN* has a broad impact on both healthy and malignant cell function. Aurora kinase A (AURKA) stabilizes MYCN through a scaffolding function independent of its kinase activity and protects it from proteolytic degradation [10]. It has been shown that AURKA inhibitors disrupt the Aurora-A/MYCN complex, triggering proteasomal degradation and resulting in decreased expression of MYCN protein, regression of tumors, and extended survival in mouse models [10–12]. Aurora kinase inhibitors have also demonstrated radio-sensitization in hepatocellular carcinoma where combination therapy with VE-465 and external beam radiation interrupted the cell cycle *in vitro* and significantly enhanced radiation-induced death *in vivo* [13].

Alisertib, a competitive reversible AURKA inhibitor, directly blocks kinase activity and disrupts the assembly of mitotic spindles, the segregation of chromosomes, and the proliferation of cells by regulating entry into mitosis [14–18]. Increased expression of AURKA, independent of *MYCN* amplification, is a negative prognostic factor in neuroblastoma, and AURKA inhibition with alisertib has shown efficacy in pre-clinical cell line xenograft models [19,20] as well as activity and safety in a pediatric Phase I trial in combination with chemotherapy [21]. Recent investigations into the role of AURKA inhibitors in the DNA damage response pathway and DNA repair have identified additional mechanisms by which AURKA inhibitors may be a promising cancer therapy [22–24].

AURKA inhibition using LY compounds has shown anti-tumor activity in pre-clinical studies [25] which has led to further investigation for a variety of solid tumors including advanced EGFR mutant non-squamous lung cancer (NCT05017025), small cell lung cancer (NCT03898791), metastatic breast cancer (NCT03955939), and relapsed/refractory neuroblastoma (NCT04106219). A phase I clinical trial studying erbumine (LY3295668) in patients with locally advanced or metastatic solid tumors showed that the drug was well tolerated overall with stable disease for nine of thirteen enrolled patients (disease control rate of 69%) [26].

Though MIBG has been studied *in vivo* using a variety of neuroblastoma and pheochromocytoma mouse models, most of these transgenic and xenograft neuroblastoma models are MIBG non-avid, possibly through loss of hNET. Only a few *in vivo* studies showing MIBG uptake have been published, including an SK-N-SH line where pinhole imaging of xenograft tumors with ^131^I-MIBG was possible [27] and a study of ultratrace MIBG in a SK-N-BE(2c) model [28]. Our current NB1691-LUC/NET mouse model is among the only published high-risk neuroblastoma mouse models which take up and retain significant and reproducible amounts of MIBG [29]. Using lentiviral transduction to exogenously express the hNET receptor in a luciferase labeled neuroblastoma cell line [30], we show that the NB1691-LUC/NET model is MIBG avid, *MYCN* amplified, and radio-resistant, and provides easy tracking of disease with bioluminescence. It is well suited for pre-clinical testing of therapies *in vivo* before these therapies are translated into children with high-risk disease.

We hypothesized that AURKA inhibition with alisertib combined with MIBG would enhance the therapeutic effect of MIBG in our MIBG avid pre-clinical model and that AURKA inhibition with either alisertib or LY3295668 would enhance radiation induced DNA damage and cell death in *MYCN* amplified neuroblastoma cell lines.

## Materials and Methods

Please see supplemental data for more detailed descriptions of the materials and methods, which have been condensed for simplicity and included here.

### Cell culture

SK-N-BE(2), Kelly, and NB1691-Luc cell lines were transduced to over express hNET as described above to enhance MIBG uptake [26]. All neuroblastoma cells were grown in DMEM media with 10% FBS, except for Kelly cells, which were grown in RPMI media with 10% FBS. Cells were maintained at 37°C in humid air with 5% CO_2_.

### Cell viability assay

Neuroblastoma cells were pre-seeded into 96-well plates for 24 hours prior to alisertib and LY3295668 treatment for 4 hours followed by external beam radiation. Cell viability was measured 72 hours post treatment using CellTiter-Glo assay. Luminescence was read on the Synergy Neo2 microplate reader.

### Immunofluorescence

Cells were pre-seeded on glass coverslips in 6-well plates. 24 hours post seeding, cells were treated with various concentrations of alisertib for either 24 hours (for G2/M cell cycle arrest) or for 4 hours followed by external beam radiation (for DNA damage analysis). Nocodazole was used as positive control to arrest cells in G2/M. Cells were fixed then permeabilized. Goat serum was used to block the cells before overnight incubation with primary antibodies. Cells were incubated with secondary antibody then mounted onto glass slides with mounting medium containing Dapi. Slides were imaged on the Leica DMi8 fluorescence microscope. Quantification of arrested cells and the number of DNA damage foci markers were performed using Fiji Image J software.

### Western blotting

Cells were lysed with RIPA lysis buffer. Western blots were performed using standard protocol.

### Flow cytometry

Kelly, SK-N-BE(2) and NB1691-LUC cells were pre-seeded in 6-well plates for 24 hours prior to alisertib treatment.

### Cell cycle arrest analysis

Cells were treated with alisertib for 4 and 24 hours then harvested and washed. Cells were fixed and permeabilized. Cells were stained with DAPI and analyzed with the BD LSR II flow cytometer. Analysis was performed using Cytobank software.

### Cell apoptosis analysis

Cells were treated with alisertib for 4 hours followed by external beam radiation. Cells were harvested 48 and 72 hours post treatment and washed and stained. Flow cytometry was performed and data was analyzed using FlowJo software.

### Immunohistochemistry

Xenograft neuroblastoma tumors, treated with alisertib and MIBG, were excised from mice and paraffin fixed for Hematoxylin and Eosin staining and analysis of cleaved caspase-3 expression using standard immunohistochemistry protocols.

### In vitro radiation

Radiation was administered via a Cesium-137 irradiator. Cells were irradiated for 1.4 minutes to receive 4 Gy.

### In vivo studies

NOD SCID gamma mice were implanted with NB1691-LUC/NET neuroblastoma cells [29]. Tumor bearing mice were treated with alisertib or saline control for 7 days via intraperitoneal (IP) injection. IP injection, which has been used previously [31,32], was used to minimize the radiation exposure of the handler while dosing. The combination and MIBG cohorts received 37 MBq (1 mCi) of ^131^I-MIBG 24 hours after the first dose of alisertib or alisertib carrier for the MIBG alone arm. Tumor size was assessed twice weekly for 25 days. Mice were euthanized once maximum tumor length reached 2.0 cm in long axis. Tumor growth was analyzed by a linear mixed effects model, similar to that described by Akutagawa et al [33]. Tumor volume, as calculated from caliper measurements, was transformed by square root to correct for heteroscedasticity and normalize residuals. Fixed effects included assigned treatment and time. Random effects were included for individual mice. Confidence intervals were estimated by the bootstrap method at the 95% level. All experiments on live vertebrates were performed in accordance with relevant institutional and national guidelines and approved by the UCSF Animal Care and Use Committee (IACUC).

## Results

### ^131^I-MIBG dosimetry: Identifying the ideal dose for combination therapy

To understand the estimated radiation dose from ^131^I-MIBG, prior studies were completed using ^124^I-MIBG as a quantitative tool for tumor imaging and dosimetry *in vivo* [29]. Several studies have estimated human-equivalent internal radiation doses using ^124^I-MIBG and PET/CT in murine xenograft models [29, 34]. From those prior results, it was estimated that administration of 52.8–206 MBq (1.43–5.57 mCi) ^131^I-MIBG delivers approximately 20 Gy radiation directly to the tumor.

**Figure 1:**
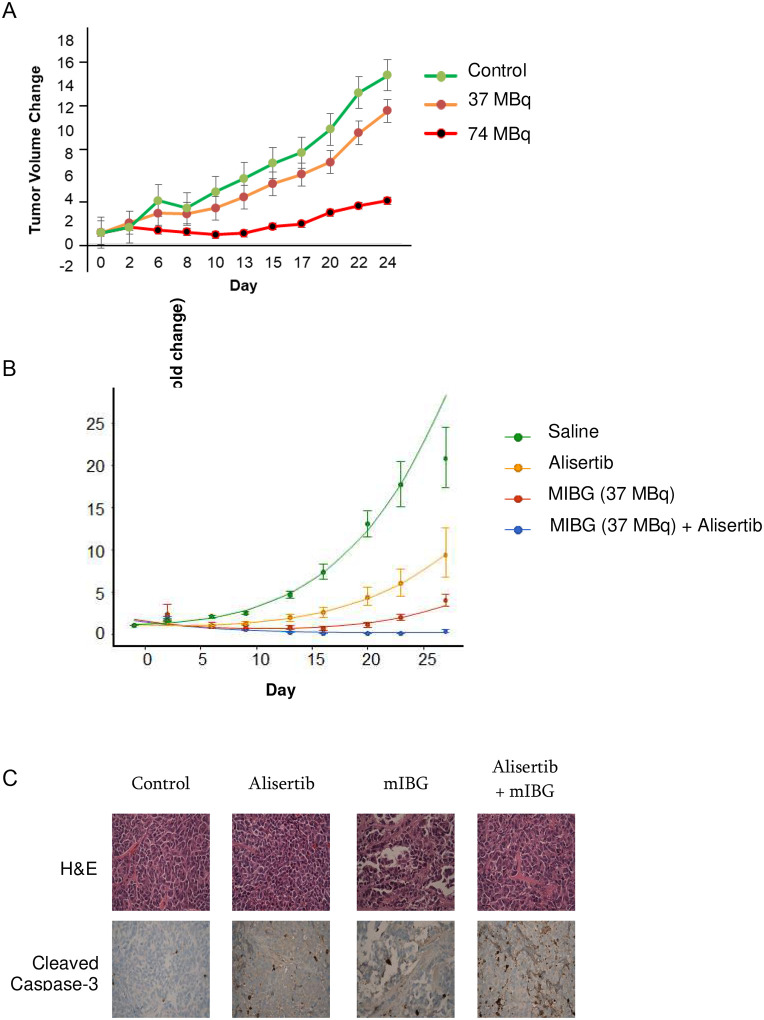
Alisertib and MIBG treatment decreases neuroblastoma tumor growth A. Identifying the ideal dose of ^131^I-MIBG for combination therapy. Mice were treated with control sham, 37 MBq, or 74 MBq. B. Alisertib and ^131^I-MIBG inhibited tumor growth and increased response to ^131^I-MIBG in NB1691-LUC/NET xenograft mice (n=5 per arm). C. Immunohistochemistry analysis showed increased cleaved caspase-3 with combination therapy. Alisertib+MIBG vs MIBG: p=0.000216; Alisertib+MIBG vs Alisertib alone: p=1.19e-05.

Using the NB1691-LUC/NET model [29], we first performed an ^131^I-MIBG dose finding experiment to determine the optimal dose for a response to ^131^I-MIBG monotherapy (Figure 1A). Treatment of NB1691-LUC/NET mouse tumors with ^131^I-MIBG alone showed decreased tumor growth for mice treated with 74 MBq (2 mCi dose), marginal therapeutic effect for mice treated with 37 MBq (1 mCi dose), and continuous tumor growth for mice with sham (saline) treatment. Overall, mice tolerated 37 MBq doses safely with good radiation induced effects on tumor growth and little systemic toxicity. We therefore chose 37 MBq for combination therapy to ensure that tumor size differences would remain evaluable with ^131^I-MIBG in combination with radiosensitizer.

### Treatment with alisertib and ^131^I-MIBG results in a significantly improved response to ^131^I-MIBG in vivo

Tumor bearing animals treated with 37 MBq (1 mCi) of ^131^I-MIBG showed a significant slowing in tumor growth as did tumors treated with alisertib alone, however the combination of alisertib and ^131^I-MIBG showed not only diminished growth, but a reduction in tumor size and an enhanced response to combination therapy (Figure 1B). Immunohistochemistry was performed which showed an increase in cleaved caspase-3 with the combination indicating an increase in apoptosis and increased cell death (Figure 1C). Mice in all cohorts tolerated therapy without significant toxicity as evaluated by weight and general well-being.

### Pretreatment with alisertib or LY3295668 followed by external beam radiation sensitizes cells to radiation and drives apoptosis

**Figure 2:**
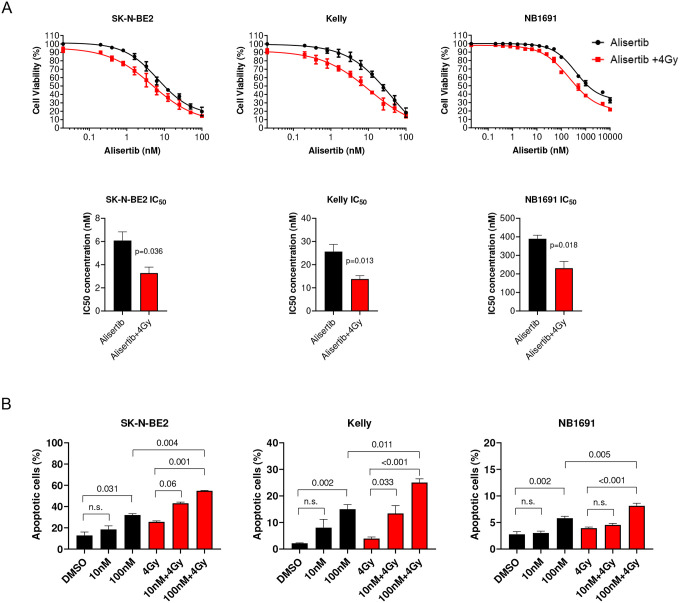
Treatment with alisertib sensitizes neuroblastoma cell lines to radiation A. Dose response of alisertib with and without radiation across three MYCN amplified cell lines pre-treated with alisertib followed by radiation showed a lower IC50 concentration; SK-N-BE(2) (p=0.036); Kelly (p=0.013); NB1691-LUC (p=0.018) (n=8 per arm). Data for IC50 concentrations were normalized to account for the effect from radiation alone. B. Flow cytometry analysis showed a significant increase in apoptosis with alisertib treatment followed by radiation.

**Figure 3:**
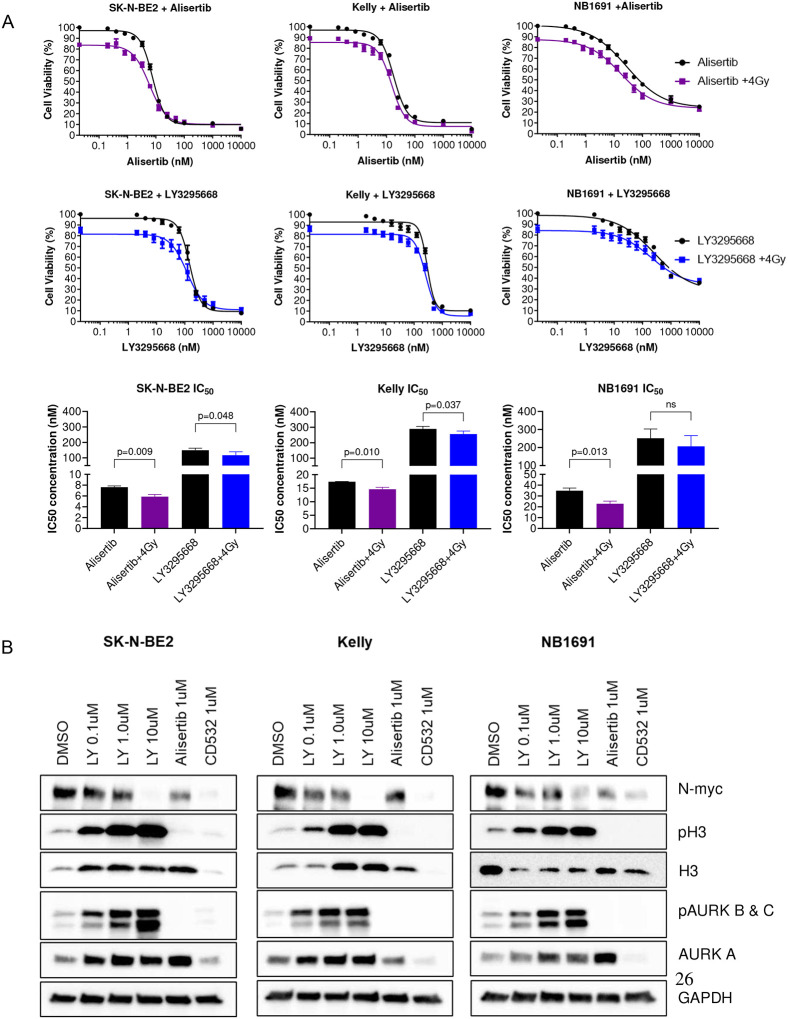
Treatment with LY3295668 sensitizes neuroblastoma cell lines to radiation A. Dose response of LY3295668 with and without radiation across three MYCN amplified cell lines pre-treated with LY3295668 followed by radiation showed a lower IC50 concentration. Data for IC50 concentrations were normalized to account for the effect from radiation alone. B. Immunoblots of cells treated with LY3295668 show that treatment increased N-myc and AURKA degradation in a dose-dependent manner.

We tested alisertib combined with external beam radiation on neuroblastoma cells derived from high-risk patients as a surrogate for MIBG therapy. Dose response testing of alisertib with and without radiation showed a leftward shift in the dose response curve indicating increased potency of the combination, manifesting as a significant decrease in the EC_50_ for alisertib (Figure 2A). A significant increase in apoptosis was observed across all three cell lines (Figure 2B). Dose response testing of LY3295668 with and without radiation also showed a leftward shift in the dose response curve, indicating increased potency of the combination, manifesting as a significant decrease in the EC_50_ for LY3295668 (Figure 3A). Increased N-myc degradation was observed across all three cell lines and this was dose-dependent (Figure 3B).

### Alisertib arrests neuroblastoma cells in G2/M

**Figure 4:**
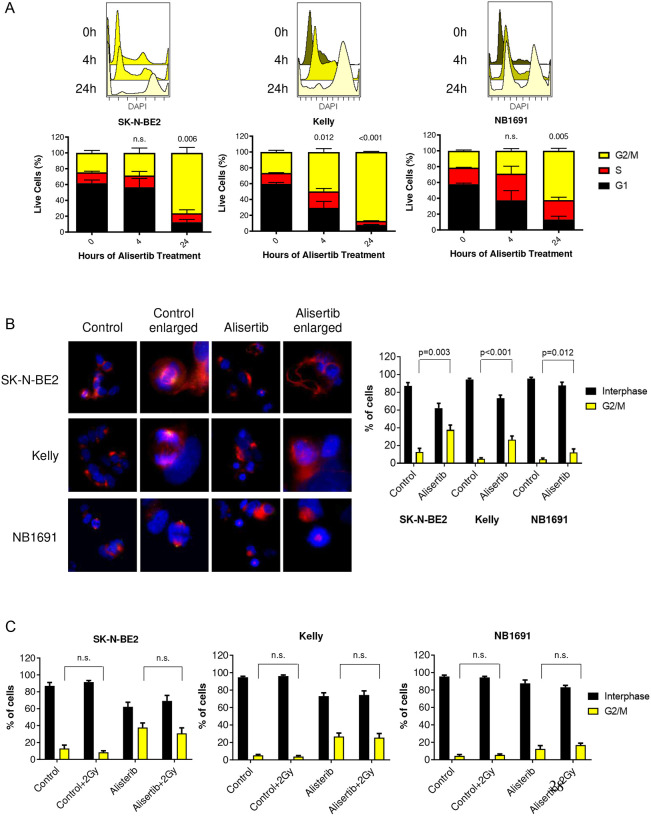
Alisertib arrests neuroblastoma cells in G2/M A. Flow cytometry analysis of neuroblastoma cells treated with alisertib showed significant increases in G2/M cell cycle arrest after 24 hours; SK-N-BE(2) (p=0.006); Kelly (p<0.001); NB1691-LUC (p=0.005). B. Immunofluorescence staining of the nucleus (blue), α-tubulin (red) and pAURKA (green) in cells treated with alisertib demonstrates uni- or multi-polar spindle formations and significant induction of cells to arrest in G2/M phase compared to non-treated cells; SK-N-BE(2) (p=0.003); Kelly (p<0.001); NB1691-LUC (p=0.012). C. γ-radiation does not impact G2/M cell cycle arrest. Data represent the % of cells arrested in G2/M.

AURKA plays a prominent role in the cell cycle with known potent effects on cell division, particularly in G2/M transition. The effects of single agent alisertib treatment on mitosis was evaluated by flow cytometry (Figure 4A) and immunofluorescence (Figure 4B). Alisertib treatment showed prominent G2/M arrest starting within 4 hours of treatment and eventually mitotic catastrophe resulting in uni- or multi-polar spindle formation. Combination treatment with alisertib and external beam radiation led to a decrease in mitotic cells, potentially during DNA repair; alisertib-induced G2/M arrest was not affected by radiation (Figure 4C). We hypothesized that the mechanism of combinatorial efficacy for AURKA inhibitors plus radiation or ^131^I-MIBG in neuroblastoma is through cell cycle arrest in G2/M, potentially allowing for open decondensed chromatin, exposing DNA to additional radiation damage and eventually leading to increased cell death by apoptosis.

### Alisertib enhances radiation induced DNA damage

**Figure 5:**
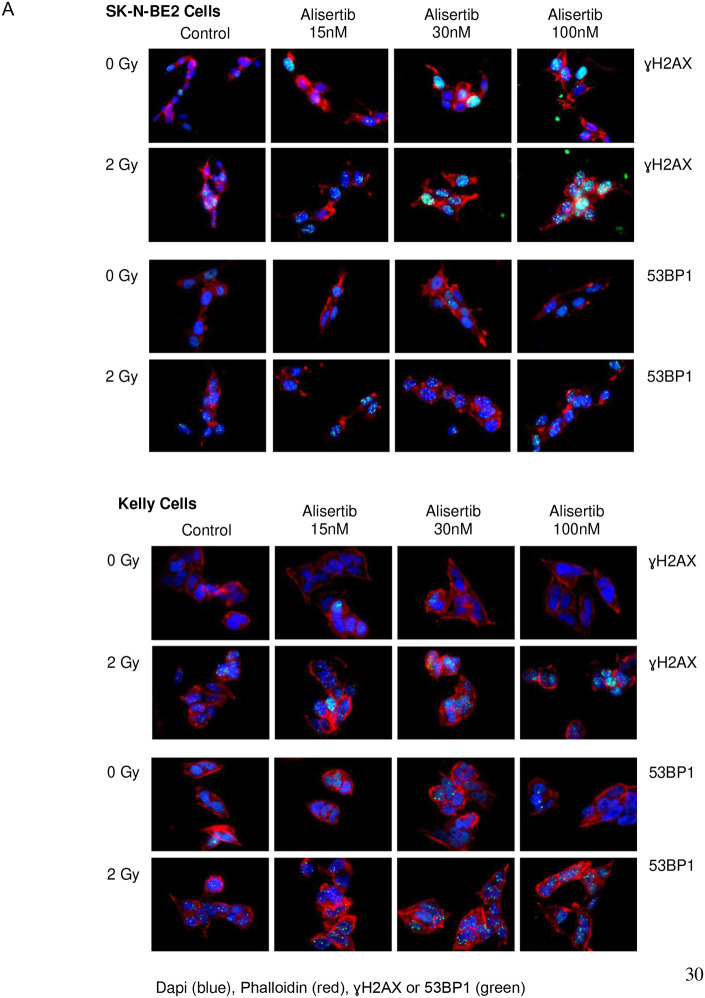

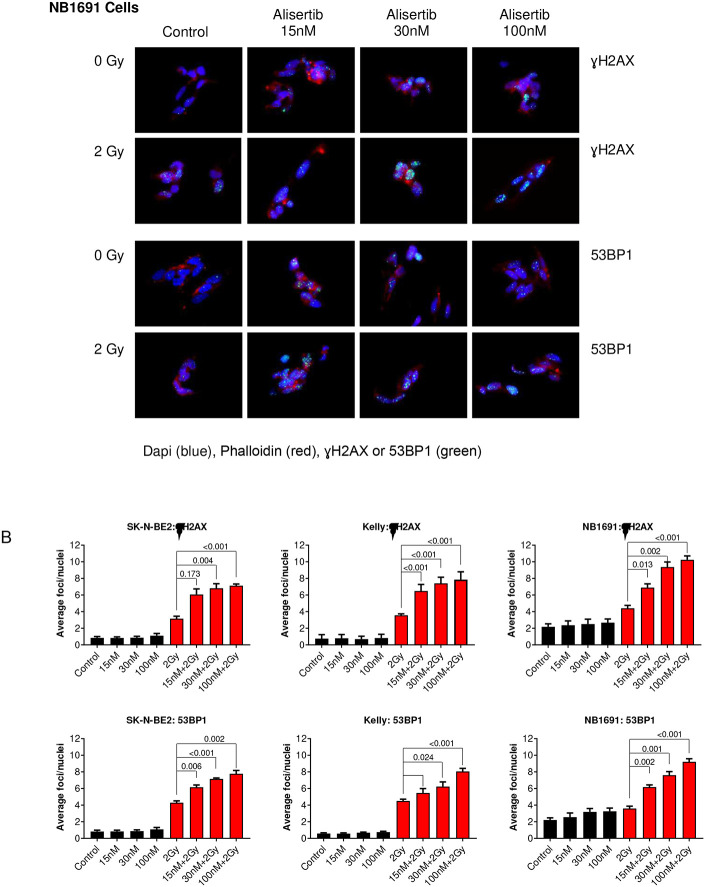
Alisertib and radiation potently increases DNA damage in neuroblastoma cells A. Cells treated with increasing doses of alisertib followed by radiation showed increased DNA damage as detected using immunofluorescence staining for γH2AX and 53BP1 foci. B. Quantitation of γH2AX and 53BP1 foci after combination treatment shows significantly greater DNA damage as exhibited by higher numbers of γH2AX and 53BP1 foci per cell across all cell lines. Blue = DAPI, green = γH2AX or 53BP1, red = Phalloidin.

Radiation induces double-stranded breaks (DSBs) in DNA which are marked by phosphorylation of the histone subtype H2AX to recruit DNA repair machinery including the p53BP1 adaptor protein. When cells are exposed to ionizing radiation or DNA-damaging agents, DSBs are generated. An early cellular response to DSBs is the rapid phosphorylation of H2AX at Ser 139 (also known as γ-H2AX). 53BP1 protein is involved in DNA-damage-checkpoint signal transduction and localizes to the sites of DNA damage after ionizing radiation. Immunofluorescence of both pH2AX and p53BP1 foci are therefore used to measure the extent of DNA damage from ionizing radiation. There is a clear increase in both pH2AX and p53BP1 foci for cells treated with 30nM alisertib and 4 Gy radiation compared to control (Figure 5A). A dose response with alisertib alone and in combination with radiation shows a clear, dose dependent, and statistically significant increase in DNA damage in the combination compared with pretreatment with alisertib alone (Figure 5B).

### Combination radiation and alisertib therapy induces apoptosis and checkpoint control pathways

**Figure 6:**
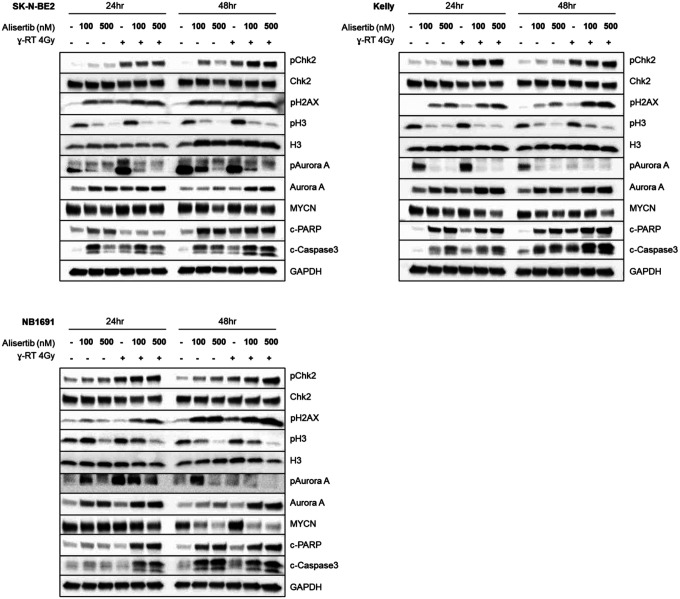
Alisertib and radiation therapy potently induces apoptosis, downregulates MYCN protein, increases DNA damage, and enhances/prolongs Chk2 activation Immunoblots of cells treated with alisertib and radiation show that treatment increases and prolongs pChk2 and pH2AX expression, decreases pH3 and pAURKA, and increases cleaved PARP and cleaved caspase-3 expression.

To better understand the mechanism of action behind the effects of combination therapy, we treated cells with alisertib 4 hours prior to 4 Gy radiation and harvested cells at 24 and 48 hours. The subsequent western blot reveals marked changes in the checkpoint kinase pathways as well as apoptosis depending on the drug tested. Alisertib shows on-target activity by decreasing AURKA auto-phosphorylation as well as by blocking Histone H3 phosphorylation across all cell lines (Figure 6). Alisertib also downregulates MYCN protein at 24 hours and shows induction of apoptotic markers cleaved PARP and cleaved caspase-3. The induction of apoptosis is notably enhanced when alisertib is combined with radiation, as measured by cleaved PARP and cleaved caspase-3. Further, radiation combined with alisertib induces sustained phosphorylation of Chk2 (a marker of ongoing DNA repair) for at least 48 hours and induces increased phosphorylation of H2AX (a marker of DNA damage) by western blot which is consistent with the immunofluorescence data in Figure 5.

## Discussion

Patients with high risk neuroblastoma and *MYCN* amplification have approximately a 40–50% overall survival, despite intensive multi-modal therapies. Thus, novel radio-sensitizing therapies are critical to improving outcomes in this patient population. Dosing, timing, and duration of drug therapy and radiation therapy can all impact the radio-sensitization of a given combination, which highlights the importance of *in vivo* testing prior to advancement to patients in clinical trials. In our study, we showed that combining an AURKA inhibitor with a targeted radiopharmaceutical markedly decreased tumor growth in vivo compared to either agent alone, and we have elucidated the mechanism of this effect. Our findings are consistent with our hypothesis that the mechanism underlying the improved efficacy of the combination of alisertib and radiation or ^131^I-MIBG is through loss of AURKA activity and subsequent blockade of cells in G2/M with enhanced DNA damage and loss of MYCN, all resulting in increased apoptosis and cell death.

Neuroblastoma is typically highly responsive to radiation which is why external beam radiation to the tumor bed after surgical resection of the primary tumor is a critical component of therapy. Though external beam radiation is effective in eliminating microscopic residual disease and minimizing the risk for relapse, it has several limitations including, most notably, the inability to target widespread metastatic disease or patchy bone marrow involvement, which can be common in refractory disease. ^131^I-MIBG, however, allows for targeted delivery of radiation to all sites of MIBG avid disease, and given its prolonged half-life, can do so for a sustained period of time (unlike external beam radiation which can only be given in limited fractions locally).

Using both a novel mouse model of MIBG avid neuroblastoma and high-risk neuroblastoma cell lines treated with radiation as a surrogate for ^131^I-MIBG, we showed that alisertib in combination with radiation enhances DNA damage and impairs/prolongs DNA repair, as evidenced by the persistence of pH2AX, a marker of double-stranded DNA breaks. It is likely that destabilization of MYCN is not the only effect of AURKA inhibitors such alisertib and LY3295668, both from these mechanistic studies and also from a Phase I trial of alisertib combined with chemotherapy in which significant responses occurred in patients whose tumors were MYCN non-amplified [21]. Other studies have suggested that alisertib treatment promotes non-homologous end joining and impairs homologous recombination. In our model systems, this hypothesis is supported by the increase in phosphorylated Chk2 expression which is reported to increase activity of BRCA1 and BRCA2, increase pH2AX, increase DNA-PKcs activity, and induce DNA double-stranded breaks [23,35]. The *in vitro* activation of checkpoint kinase pathways and cell cycle arrest further suggests that combination therapy halts natural progression through the cell cycle, likely due to checkpoint regulation and the inability of the cell to properly repair damaged DNA.

Limitations of this research include the fixed dosing and schedule of therapy administration *in vivo* as well as the evaluation of mechanism only *in vitro* using MYCN-amplified cell lines. ^131^I-MIBG is thought to work by accumulating in clusters of neuroblastoma tumor cells and within each neuroblastoma cell serving as a mini-irradiator for its neighboring cells, making *in vivo* experiments reflective of human tumors; however because of the significant logistical barriers to performing molecular testing on live cells in radioactive tumor-bearing mice, we elected to use cell line models of high-risk neuroblastoma to further elucidate the mechanism.

## Conclusion

We have demonstrated that AURKA inhibition by alisertib or LY3295668 can sensitize highly radio-resistant neuroblastoma tumors and cell lines to selective radiation therapy with ^131^I-MIBG or radiation and that this combination therapy is active. These data provide proof of principle for the use of our NB1691-LUC/NET pre-clinical model for testing new combinations with ^131^I-MIBG and support further testing of AURKA inhibitors with ^131^I-MIBG in children with high-risk neuroblastoma in future clinical trials. Enabled by this work, future studies will allow the head-to-head pre-clinical comparison of different radiosensitizers with ^131^I-MIBG to optimize therapies prior to initiating toxic and costly clinical trials in children with neuroblastoma.

## Data Availability

The datasets used and/or analyzed during the current study are available from the corresponding author on reasonable request.

## List of abbreviations

MIBG: ^131^I-metaiodobenzylguanidine
AURKA: Aurora kinase A
hNET: human norepinephrine transporter
HDAC: histone deacetylase
DSBs: double-stranded breaks
